# microRNA-146a promotes mycobacterial survival in macrophages through suppressing nitric oxide production

**DOI:** 10.1038/srep23351

**Published:** 2016-03-30

**Authors:** Miao Li, Jinli Wang, Yimin Fang, Sitang Gong, Meiyu Li, Minhao Wu, Xiaomin Lai, Gucheng Zeng, Yi Wang, Kun Yang, Xi Huang

**Affiliations:** 1 Program of Immunology, Affiliated Guangzhou Women and Children’s Medical Center, Zhongshan School of Medicine, Sun Yat-sen University, Guangzhou 510080, China; 2 Institute of Tuberculosis Control, Key Laboratory of Tropical Diseases Control (Sun Yat-sen University), Ministry of Education, Guangzhou 510080, China; 3 Institute of Molecular Immunology, School of Biotechnology, Southern Medical University, Guangzhou 510515, China; 4 Guangzhou Chest Hospital, Guangzhou, 510095, China

## Abstract

Macrophages play a crucial role in host innate anti-mycobacterial defense, which is tightly regulated by multiple factors, including microRNAs. Our previous study showed that a panel of microRNAs was markedly up-regulated in macrophages upon mycobacterial infection. Here, we investigated the biological function of miR-146a during mycobacterial infection. miR-146a expression was induced both *in vitro* and *in vivo* after *Mycobacterium bovis* BCG infection. The inducible miR-146a could suppress the inducible nitric oxide (NO) synthase (iNOS) expression and NO generation, thus promoting mycobacterial survival in macrophages. Inhibition of endogenous miR-146a increased NO production and mycobacterial clearance. Moreover, miR-146a attenuated the activation of nuclear factor κB and mitogen-activated protein kinases signaling pathways during BCG infection, which in turn repressed iNOS expression. Mechanistically, miR-146a directly targeted tumor necrosis factor (TNF) receptor-associated factor 6 (TRAF6) at post-transcriptional level. Silencing TRAF6 decreased iNOS expression and NO production in BCG-infected macrophages, while overexpression of TRAF6 reversed miR-146a-mediated inhibition of NO production and clearance of mycobacteria. Therefore, we demonstrated a novel role of miR-146a in the modulation of host defense against mycobacterial infection by repressing NO production via targeting TRAF6, which may provide a promising therapeutic target for tuberculosis.

Tuberculosis (TB) is still a leading public health threat worldwide with high morbidity and mortality^1^. In 2014, World Health Organization reported that an estimated 9.0 million incident cases of TB occurred, with 1.5 million deaths caused by the disease^2^. *Mycobacterium tuberculosis* (MTB) is the causative agent responsible for TB, and infects approximately one-third of the human population globally. However, only about 10% of infected individuals develop active TB, while the remaining 90% cases exhibit latent infection, indicating a critical role of the host immunity in the containment of MTB infection^3^.

Macrophages act as the first line of host immune defense against MTB^4^. The infectious bacilli are inhaled as aerosol particles, and phagocytosed by resident macrophages. Invading MTB are recognized by macrophages through pattern-recognition receptors (PRRs), which trigger innate immune defense, and subsequently initiate adaptive immune responses to pathogenic MTB^5^,^6^,^7^. During TB infection, Toll-like receptors (TLRs) are involved in host innate recognition of MTB^8^,^9^,^10^,^11^. Engagement with cognate ligands activates the TLR signaling pathway via the adaptor myeloid differentiation primary response gene 88 (MyD88)^12^. Sequentially, tumor necrosis factor (TNF) receptor-associated factor 6 (TRAF6) relays MyD88-dependent TLR signaling and leads to the activation of downstream nuclear factor kappa B (NF-κB) and mitogen-activated protein kinases (MAPKs, including JNK, ERK and p38) pathways^13^, which ultimately results in the production of inflammatory cytokines and direct antimicrobial mediators, such as TNF and nitric oxide (NO)^14^.

The role of NO in macrophage-mediated anti-mycobacterial defense has been well characterized in mouse model of TB^7^,^15^. In macrophages, NO is generated by inducible nitric oxide synthase (iNOS)-mediated metabolism of amino acid L-arginine^16^. Several lipoproteins from MTB stimulate TLR-dependent transcription of iNOS and subsequent production of NO in macrophages, which kills intracellular MTB directly^17^,^18^. Deficiency of iNOS renders mice highly susceptible to MTB infection with high mycobacterial burden and decreased survival^19^. In addition, inhibition of iNOS in human alveolar macrophages from TB patients abolishes the macrophage-mediated anti-mycobacterial activity *in vitro*^20^. These studies establish a critical role of iNOS-mediated NO production in host defense against MTB. In macrophages, the expression of iNOS is mainly regulated at the transcriptional level^15^. Microbial products, pro-inflammatory cytokines and interferons are prototypical inducers of iNOS^15^. These factors trigger NF-κB, MAPK or signal transducer and activator transcription 1 (STAT1) pathways, which initiate the transcription of iNOS gene in macrophages^16^,^21^. Nevertheless, whether microRNAs (miRNAs) regulate iNOS expression and NO production during innate immune response remains largely unknown.

miRNAs are small non-coding RNAs that control gene expression in diverse biological processes, such as proliferation, differentiation and immune response^22^. Through targeting 3′UTR of mRNA, miRNAs result in degradation of transcripts or repression of translation^23^. To date, several miRNAs have been reported to participate in the regulation of host immunity to MTB infection^24^. Our previous study reveals that several miRNAs are induced in macrophages during MTB infection, including miR-155, miR-146a and miR-132^25^. Mycobacteria-induced miR-155 targets Ras homolog enriched in brain (Rheb) to enhance autophagy in macrophages, which, in turn, promotes elimination of intracellular bacilli^26^. In lymphocytes, miR-29 has been reported to be a suppressor of IFN-γ production by directly targeting its mRNA, thus facilitating the survival of MTB and other intracellular pathogens^27^. In patients with active TB, miR-582-5p is up-regulated and reduces MTB-induced apoptosis of monocytes by targeting FOXO1^28^. However, it remains unclear whether mycobacteria-triggered miRNAs regulate NO-mediated bacterial clearance.

In the present study, we sought to investigate the potential role of miR-146a in host immune defense against mycobacteria. Our data demonstrated, for the first time, that mycobacteria-induced miR-146a repressed iNOS expression and NO production in macrophages, which facilitated mycobacterial survival. Moreover, miR-146a suppressed NF-κB and MAPKs pathways by targeting TRAF6, thus inhibiting iNOS expression. These findings unravel a novel regulatory mechanism of anti-mycobacterial response by miRNA, and may provide a promising therapeutic target for TB.

## Results

### *M. bovis* BCG infection induces miR-146a expression both *in vitro* and *in vivo*

**Figure 1:**
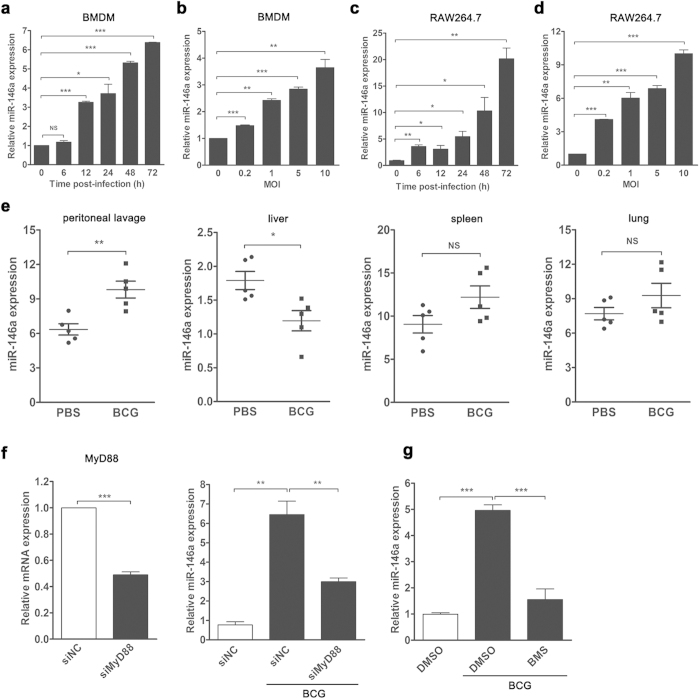
The expression of miR-146a were up-regulated after mycobacterial infection both *in vivo* and *in vitro*. Murine BMDMs (**a**,**b**) or RAW264.7 cells (**c**,**d**) were infected with *M. bovis* BCG at an MOI of 5 for the indicated time (**a**,**c**) or at the indicated MOIs for 24 h (**b**,**d**). Peritoneal lavage fluid and different organs were collected from BCG-infected or PBS-treated mice (n = 5).The expression levels of miR-146a were measured by real-time PCR (**e**). RAW264.7 cells were transfected with siRNA targeting MyD88 (**f**) or pretreated with IKKα/β inhibitor (BMS345541) (**g**), followed by BCG infection. The mRNA levels of MyD88 and miR-146a were measured by real-time PCR (**f**,**g**). Data are shown as mean ± s.e.m. of three independent experiments. *p < 0.05; **p < 0.01; ***p < 0.001; NS, no significance.

To explore the expression of miR-146a during mycobacterial infection, murine primary BMDMs and macrophage-like cell line RAW264.7 were challenged with *M. bovis* BCG. Real-time PCR was used to determine the expression level of miR-146a. We found that the expressions of miR-146a in BCG-infected BMDMs increased in a time- and dose-dependent manner (Fig. 1a,b). The level of miR-146a in BMDMs at 72 h post-infection was about 6-fold higher than that in uninfected control (Fig. 1a). Similarly, miR-146a expressions were elevated in RAW264.7 cells after BCG challenge (Fig. 1c,d). More than 20-fold increase in miR-146a expression was observed in BCG-challenged RAW264.7 cells at 72 h post-infection compared to uninfected cells (Fig. 1c). Together, these results indicated that BCG infection induced miR-146a expression in murine macrophages.

To further determine the expression pattern of miR-146a after mycobacterial infection *in vivo*, we challenged C57BL/6 mice with *M. bovis* BCG intraperitoneally for 7 days, and tested miR-146a expression in lungs, spleens, livers and peritoneal lavage fluid with real-time PCR. The expression of miR-146a was up-regulated in peritoneal lavage fluid and down-regulated in livers of BCG-infected mice, while no significant increase was detected in either spleens or lungs (Fig. 1e). These results suggested induction of miR-146a in local infection site but not in remote organs.

Moreover, we investigated the signaling pathway involved in miR-146a induction during mycobacterial infection. The adaptor MyD88 of TLRs is critical for innate recognition of invading mycobacteria^29^. Therefore, we knocked down MyD88 with RNAi technique and examined the expression level of mycobacteria-induced miR-146a. Silencing MyD88 expression decreased miR-146a expression by 50% approximately (Fig. 1f). Since previous study revealed transcription factor NF-κB binding site in the promoter of miR-146a^30^, we next tested the hypothesis that NF-κB mediated the transcription of miR-146a after BCG infection. Pharmacological inhibition of NF-κB signaling reduced miR-146a expression in mycobacteria-challenged macrophages (Fig. 1g). These results suggested that the induction of miR-146a during mycobacterial infection was dependent on MyD88-NF-κB signaling pathway.

### miR-146a promotes mycobacterial survival in macrophages

**Figure 2:**
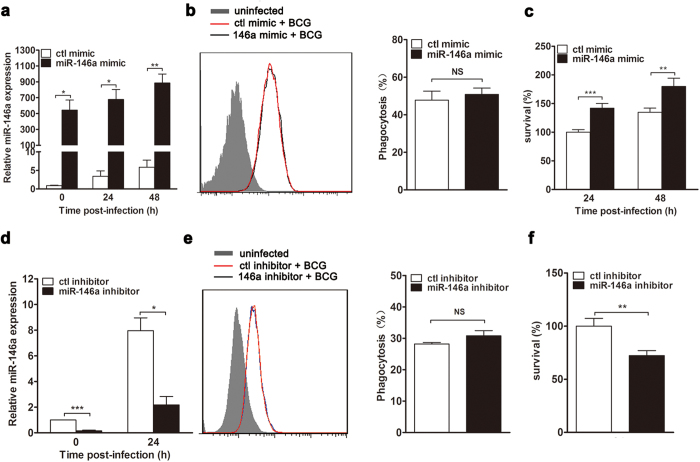
miR-146a promotes the survival of *M. bovis* BCG in macrophages. RAW264.7 cells were transfected with miR-146a mimic (**a**–**c**) or inhibitor (**d**–**f**) for 24 h, followed by BCG infection. The relative expression levels of miR-146a were determined by real-time PCR (**a**,**d**). Phagocytosis of Texas Red-labeled BCG was detected by flow cytometry (**b**,**e**). Mycobacterial viability was determined by CFU assay, and the survival was expressed as a percentage of the control (**c**,**f**). Data are shown as mean ± s.e.m. of three independent experiments. *p < 0.05; **p < 0.01; ***p < 0.001; NS, no significance.

To investigate the role of miR-146a in host defense against mycobacterial infection, macrophages were overexpressed with chemically synthetic miR-146a mimic, and the phagocytosis and the survival of mycobacteria were examined. RAW264.7 cells were transiently transfected with control or miR-146a mimic, followed by BCG infection. Real-time PCR showed that miR-146a levels increased dramatically after mimic transfection, confirming the efficacy of overexpression (Fig. 2a). miR-146a mimic-transfected RAW264.7 cells were challenged with Texas Red-labeled BCG, and flow cytometry was performed to measure the phagocytosis of mycobacteria in macrophages. Our results showed that overexpression of miR-146a had no major effect on the phagocytosis of BCG (Fig. 2b). Furthermore, to determine whether miR-146a regulates the clearance of mycobacteria in macrophages, miR-146a mimic-transfected RAW264.7 cells were challenged with BCG, and the viability of BCG was tested by colony-forming unit (CFU) assay. CFU data showed that the survival of BCG increased in miR-146a-overexpressed RAW264.7 cells at 24 h and 48 h post-infection (Fig. 2c), suggesting an inhibitory role of miR-146a in host defense against mycobacteria.

Moreover, inhibitor was used to antagonize endogenous miR-146a to validate its role in mycobacterial infection. When specific inhibitor was transfected into RAW264.7 cells, the level of induced miR-146a was dramatically reduced after BCG challenge (Fig. 2d). Consistent with mimic results above, inhibition of miR-146a enhanced mycobactericidal activity of RAW264.7 cells (Fig. 2f), while had no effect on phagocytosis of mycobacteria (Fig. 2e). Collectively, these results suggested that miR-146a decreased macrophage-mediated killing of mycobacteria.

### Mycobacteria-induced miR-146a impairs iNOS expression and NO production by inhibiting NF-κB and MAPKs pathways

**Figure 3:**
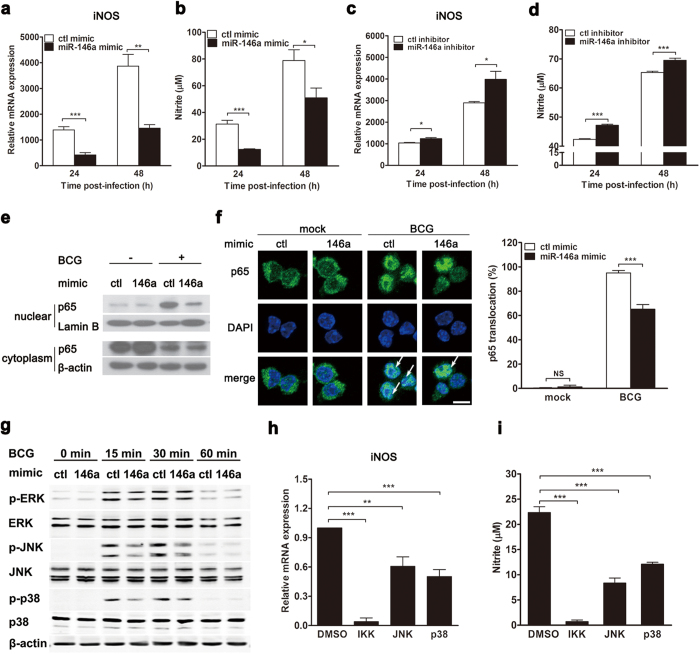
Mycobacteria-induced miR-146a impairs iNOS expression and NO production by inhibiting NF-κB and MAPKs pathways. RAW264.7 cells were transfected with miR-146a mimic or inhibitor for 24 h, followed by BCG infection (**a**–**d**). The mRNA levels of iNOS (**a**,**c**) and NO production (**b**,**d**) were measured by real-time PCR and Griess assay, respectively. The protein level of NF-κB p65 subunit in nucleus was detected by Western blot (**e**) and confocal microscopy (**f**). Arrows indicate NF-κB p65 accumulation in nucleus (**f**). Scale bar, 5 μm. The phosphorylation of ERK, JNK and p38 were determined by Western blot (**g**). RAW264.7 cells were pretreated with inhibitors for IKK, JNK or p38 for 1 h, followed by BCG infection. The mRNA levels of iNOS (**h**) and NO production (**i**) were measured by real-time PCR and Griess assay, respectively. Data are shown as mean ± s.e.m. of three independent experiments. *p < 0.05; **p < 0.01; ***p < 0.001, NS, no significance.

To elucidate the underlying mechanism for miR-146a-mediated inhibition of mycobactericidal activity in macrophages, we tested the effect of miR-146a on iNOS expression and NO production, which is a well-characterized anti-mycobacterial mechanism. RAW264.7 cells were transfected with control or miR-146a mimic followed by BCG challenge, and the expression of iNOS and the production of NO were examined by real-time PCR and Griess assay, respectively. Overexpression of miR-146a mimic reduced iNOS mRNA expression (Fig. 3a) and NO production (Fig. 3b) in BCG-challenged RAW264.7 cells. Consistently, when endogenous miR-146a was inhibited, both iNOS mRNA level and NO production increased in RAW264.7 cells (Fig. 3c,d). In addition, autophagy pathway was examined in miR-146a-transfected macrophages, which is another antimicrobial mechanism during mycobacterial infection. We found that miR-146a had no major effect on autophagy (data not shown).

To determine the mechanism by which miR-146a suppressed iNOS expression, we examined the activation of upstream NF-κB and MAPKs (including ERK, JNK and p38) pathways. Resting NF-κB distributes in cytoplasm, while activated NF-κB subunits form dimers and translocate into nucleus to initiate the transcription of target genes. Therefore, we extracted proteins from nuclei and cytoplasm, and examined the protein levels of NF-κB p65 subunit in both compartments to determine the activation of the pathway. Western blot result showed that miR-146a inhibited nuclear accumulation of NF-κB p65, suggesting inhibition of NF-κB activation (Fig. 3e). Moreover, we observed attenuated nuclear translocation of NF-κB p65 in miR-146a-overexpressed cells using confocal microscopy (Fig. 3f). The activation of the MAPKs signaling pathways in miR-146a-transfected cells was detected by Western blot after BCG infection. The result showed that the phosphorylation of JNK and p38 in BCG-infected RAW264.7 cells were decreased after miR-146a mimic transfection (Fig. 3g). However, there was no major difference in ERK phosphorylation between cells transfected with control and miR-146a mimics (Fig. 3g). Furthermore, iNOS expression and NO production were examined in BCG-challenged macrophages when NF-κB and MAPK signaling were blocked with small molecule inhibitors. We found that inhibition of IKKα/β, JNK or p38 MAPKs in macrophages reduced both iNOS expression (Fig. 3h) and NO production (Fig. 3i) after BCG infection. Notably, blocking NF-κB pathway with IKKα/β inhibitor decreased NO production by 90%, suggesting a dominant role of NF-κB pathway in NO production during mycobacteria infection. Collectively, these data suggested that miR-146a impaired BCG-induced iNOS expression and NO production in macrophages by inhibiting NF-κB, JNK and p38 MAPKs pathways.

### miR-146a represses TRAF6 expression post-transcriptionally to inhibit iNOS expression

**Figure 4:**
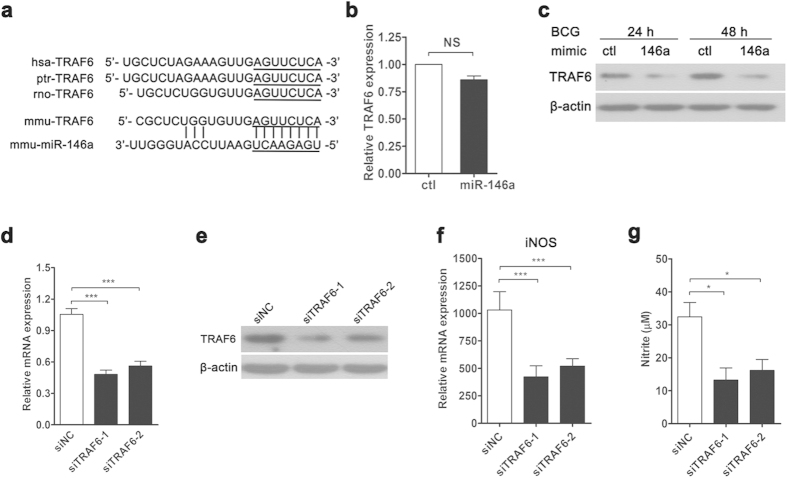
miR-146a represses TRAF6 expression post-transcriptionally to inhibit iNOS expression. Sequence of miR-146a and its predicted binding with the TRAF6 3′UTRs of different species are shown (**a**). The mRNA (**b**) and protein (**c**) levels of TRAF6 in control or miR-146a mimic-transfected RAW264.7 cells were measured by real-time PCR and Western blot, respectively. RAW264.7 cells were transfected with negative control siRNA (siNC), TRAF6 siRNA-1or siRNA-2, followed by BCG infection for 24 h. The mRNA and protein levels of TRAF6 were examined (**d**,**e**). The mRNA expression level of iNOS (**f**) and the nitrite level (**g**) were measured by real-time PCR and Griess assay, respectively. Data are shown as the mean ± s.e.m. of three independent experiments. *p < 0.05; ***p < 0.001, NS, no significance.

To identify the specific target of miR-146a that modulated NO production, bioinformatics analysis was performed with TargetScan (http://www.targetscan.org). We found that TRAF6, which locates upstream of NF-κB and MAPKs pathways, displayed a potential evolutionarily-conserved seed match for miR-146a in its 3′UTR (Fig. 4a). Previous study reported that virus-induced miR-146a targeted TRAF6, and negatively regulated antiviral pathway^31^. To confirm whether miR-146a targeted TRAF6 during mycobacterial infection, we examined TRAF6 expression in RAW264.7 cells transfected with control or miR-146a mimic. As expected, miR-146a did not alter mRNA level of TRAF6 (Fig. 4b), but substantially reduced TRAF6 protein levels at 24 h and 48 h post-infection (Fig. 4c).

To further determine whether TRAF6 was directly involved in regulating iNOS expression and NO production during BCG infection, we silenced TRAF6 expression with siRNA in RAW264.7 cells. Two siRNAs with different target sequences were used to knockdown TRAF6 expression, and the efficacy of each siRNA was confirmed with both real-time PCR (Fig. 4d) and Western blot (Fig. 4e). Silencing TRAF6 decreased iNOS expression in BCG-challenged RAW264.7 cells (Fig. 4f). In accordance, TRAF6-knockdown cells produced less NO upon BCG infection (Fig. 4g). Together, our data suggested that BCG-induced miR-146a suppressed TRAF6 expression, which resulted in impairment of iNOS expression and NO production.

### Overexpression of TRAF6 reverses miR-146a-mediated inhibition of NO production and clearance of mycobacteria

**Figure 5:**
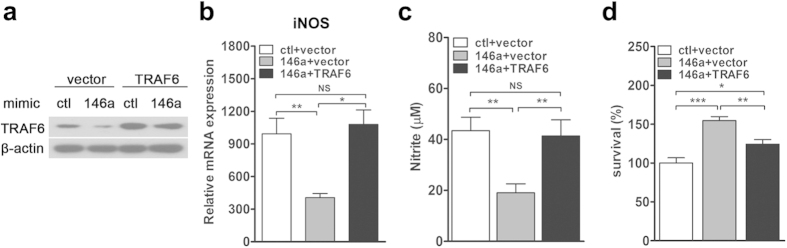
Overexpression of TRAF6 reverses miR-146a-mediated inhibition of NO production and BCG clearance. RAW264.7 cells were co-transfected with control or miR-146a mimic together with empty vector or TRAF6 plasmid for 24 h. The expression levels of TRAF6 were determined by Western blot (**a**). RAW264.7 cells were co-transfected with mimics and plasmids as described above, followed by BCG infection for 24 h. The mRNA expression level of iNOS (**b**) and the nitrite level in the culture supernatant (**c**) were measured by real-time PCR and Griess assay, respectively. Mycobacterial viability was determined by CFU assay, and survival was expressed as a percentage of the control (**d**). Data are shown as the mean ± s.e.m. of three independent experiments. *p < 0.05; **p < 0.01; ***p < 0.001, NS, no significance.

Furthermore, we explored whether overexpression of TRAF6 could restore NO production and BCG clearance in miR-146a-overexpressed macrophages. RAW264.7 cells were co-transfected with control or miR-146a mimics, together with empty vector or TRAF6 plasmid. Western blot data showed that overexpression of miR-146a decreased TRAF6 expression, but co-transfection of TRAF6 plasmid recovered its expression (Fig. 5a). Consistent with data above, both iNOS expression and NO production were suppressed by miR-146a in RAW264.7 cells co-transfected with empty vector (Fig. 5b,c). However, when TRAF6 were overexpressed, miR-146a failed to inhibit either iNOS expression or NO production (Fig. 5b,c). CFU assay showed that the viability of BCG was increased in miR-146a-transfected RAW264.7 cells (Fig. 5d). Nevertheless, overexpression of TRAF6 abolished miR-146a-mediated enhancement of mycobacterial survival in macrophages (Fig. 5d). Together, these data indicated that BCG-induced miR-146a targeted TRAF6 to repress iNOS expression and NO production, thus increased the survival of BCG in macrophages.

## Discussion

miRNAs have been demonstrated to play an essential role in host response to intracellular mycobacteria^32^. Nevertheless, whether miRNAs regulate NO-mediated anti-mycobacterial defense remains largely unknown. The present study reveals a novel role of miR-146a in regulating iNOS expression and NO production in macrophages upon *M. bovis* BCG infection. Our data show that miR-146a impairs NF-κB, JNK and p38 MAPKs signaling activation by targeting TRAF6, resulting in an inhibition of NO production and BCG killing. These findings provide a better understanding of miR-146a in regulation of host innate defense against mycobacteria.

Emerging evidence has shown that miR-146a expresses in multiple cell types, and closely relates to inflammation, antiviral response and adaptive immunity^33^. Several microbial infections can up-regulate miR-146a expression. Either Dengue virus or Enterovirus 71 infection can induce significant increase in miR-146a expression in human monocytes^34^,^35^. Moreover, a previous study shows that miR-146a expression is up-regulated in macrophages after *Listeria monocytogenes* infection^36^. In this study, we show that miR-146a expression is enhanced dramatically after *M. bovis* BCG infection in murine primary macrophages and macrophage-like cell line in a time- and dose-dependent manner. Previous miRNA microarray studies reveal altered expression of miR-146a during MTB infection. For instance, miR-146a expression is down-regulated in mononuclear cells obtained from peripheral blood of TB patients^37^. The discrepancy in miR-146a expression between our findings in macrophages and others in peripheral blood mononuclear cells (PBMCs) may be due to difference in cell types, since PBMCs are composed of T cells, B cells, NK cells, monocytes, etc. These immune cells probably exhibit different expression pattern of miR-146a during mycobacterial infection, which needs further investigation. In line with this notion, mycobacteria-infected mice in our study showed no increase in miR-146a expression in splenocytes which also include various cell types. Liu *et al.* have reported that the expression of miR-146a decreases in alveolar macrophages of TB patients, but increases in *M. bovis* BCG-infected THP-1 cells^38^. These data revealed different expression patterns of miR-146a between *ex vivo* and *in vitro* macrophages. While bacilli stimulate macrophages directly during *in vitro* challenge, *ex vivo* alveolar macrophages isolated from bronchial lavage fluid might not be invaded by MTB. In the later setting, the expression of miR-146a in alveolar macrophages may be regulated by lung microenvironment rather than triggered by bacilli.

The critical role of miR-146a in modulation of innate immunity has been extensively investigated. For instance, virus-induced miR-146a negatively modulates retinoic acid inducible gene I (RIG-I)-like receptor pathway, thus impairs innate antiviral response^31^. miR-146a suppresses TLR signaling and is critical for LPS-induced tolerance in monocytic THP-1 cells^39^. Aberrant expression of miR-146a results in the age-associated dysfunction of macrophages, which loss responsiveness to LPS^40^. Nevertheless, the function of miR-146a during bacterial infection remains ill-defined. Recently, Li *et al.* demonstrate that miR-146a increases mycobacterial replication by decreasing pro-inflammatory cytokines production in murine macrophages^41^. Another study also shows that miR-146a attenuate *M. bovis* BCG-induced TNF-α production in human monocytic THP-1 cells^38^. In our study, we find that miR-146a facilitates the survival of BCG by inhibiting the NO generation in macrophages. Therefore, miR-146a functions in distinct signal pathways and exerts diverse effects in macrophages during mycobacterial infection.

Due to diversity of targets, several lines of evidence supports that miR-146a is a multifunction molecule in regulating immune response. For example, miR-146a is prevalently expressed in regulatory T cells, and is critical for their suppressive activity on Th1 responses by targeting STAT1^42^. During inflammatory response, miR-146a inhibits NF-κB RelB subunit expression and controls the amplitude of the Ly-6C^hi^ monocyte response^43^. Moreover, miR-146a acts as a negative regulator of TLR signaling by targeting TRAF6 and IRAK^44^. Consistently, our findings indicate that miR-146a inhibits expression of TRAF6 at post-transcriptional level, and modulates the iNOS expression and NO production during mycobacterial infection. While TRAF6 overexpression abolished miR-146a-induced suppression of iNOS expression and NO generation, miR-146a-mediated inhibition of mycobacterial clearance was not completely reversed by TRAF6 overexpression. This finding suggests that miR-146a may play a minor antimycobacterial role independent of TRAF6-mediated NO production. In accordance with this notion, a recent study revealed that miR-146a could target prostaglandin-endoperoxide synthase 2 (PTGS2) to disrupt the killing of intracellular mycobacteria^38^. Therefore, miR-146a negatively regulates host defense against bacterial infection by targeting various genes.

The NO production is precisely regulated by several factors at both transcriptional and post-transcriptional levels^15^. Recent studies reveal fine-tuning of iNOS expression by miRNAs. miR-939 and miR-26a are reported to directly interact with the 3′-UTR of iNOS mRNA in hepatocytes^45^ and T cell lymphoma^46^, respectively. In LPS-stimulated murine macrophages, miRNA-155 negatively regulates suppressor of cytokine signaling 1 (SOCS1) expression, leading to enhancement of STAT1 signaling and up-regulation of the iNOS expression indirectly^47^. Previously, macrophages from TRAF6-deficient mice have been reported to fail to induce the activation of NF-κB and MAPKs pathways and the expression of pro-inflammatory cytokines in response to TLR ligands stimulation^13^. Besides, during mycobacterial infection, NF-κB and MAPKs signaling pathways mediate induction of iNOS expression. In accordance, we show that TRAF6 expression is inhibited by miR-146a, which in turn impairs NF-κB and MAPKs signaling activation, thus negatively regulating iNOS gene expression indirectly.

In summary, the present study indicates that miR-146a is induced in macrophages in response to mycobacterial infection. Inducible miR-146a attenuates iNOS expression and NO production in macrophages by targeting TRAF6, thus dampening host defense against intracellular bacteria. Our study unravels a crucial role of miR-146a in NO generation and mycobacterial elimination, which may provide better understanding of the pathogenesis of TB and useful information for developing potential therapeutic interventions against the disease.

## Methods

### Ethics statement

All experimental protocols were approved by Sun Yat-sen University. The methods used in this study were carried out in accordance with the approved guidelines. All animal experiments were approved by the Animal Ethics Committee of Sun Yat-sen University and performed in accordance with the guidelines of Animal Care and Use of Sun Yat-sen University.

### Reagents

Middlebrook 7H10 agar and Middlebrook 7H9 broth medium were purchased from BD Difco Laboratories (Sparks, MD). Texas Red dye was obtained from Invitrogen (Carlsbad, CA). Antibodies against NF-κB p65 (sc-109), Lamin B (sc-6216), JNK (sc-571) and p38 (sc-7149) were obtained from Santa Cruz Biotechnology (Santa Cruz, CA). Monoclonal (ab33915) and polyclonal (sc-7221) antibodies against TRAF6 were from Abcam and Santa Cruz Biotechnology, respectively. Antibodies against ERK1/2 (#4695), phosphorylated ERK1/2 (#4370), phosphorylated JNK (#4668) and phosphorylated p38 (#9215) were obtained from Cell Signaling Technology (Beverly, MA). The β-actin (A1978) antibody was obtained from Sigma-Aldrich (St. Louis, MO). Alexa Fluor 488-conjugated Goat Anti-Rabbit IgG and ProLong Gold antifade reagent were from Invitrogen. Inhibitors of IKKα/β (BMS345541), JNK (SP600125) and p38 (SB203580) were obtained from Merck (Darmstadt, Germany).

### Cells and *M. bovis* BCG culture

Murine macrophage-like RAW264.7 cells were maintained in DMEM supplemented with 10% fetal bovine serum (FBS), 1 mM sodium pyruvate, 100 U/ml penicillin and 100 mg/ml streptomycin (GIBCO). Murine bone marrow-derived macrophages (BMDMs) were prepared from bone marrow cells from femurs and tibias of 6- to 8-week-old C57BL/6 mice as described before^25^,^48^. All animal experimental procedures were approved by the Medical Ethics Committee and Biosafety Management Committee of Sun Yat-sen University. *M. bovis* BCG strain 19015 was purchased from the American Type Culture Collection (ATCC), and were grown in Middlebrook 7H9 broth medium supplemented with 10% OADC and cultured in a standard culture incubator as reported before^49^.

### *M. bovis* BCG infection of mice

Female 6- to 8-week-old C57BL/6 mice were injected with 1 × 10^6 ^CFU *M. bovis* BCG or PBS intraperitoneally. Mice were sacrificed at day 7 post-infection, and peritoneal lavage fluid and organs (lung, spleen and liver) were collected for RNA isolation.

### Colony-forming unit (CFU) assay

RAW264.7 cells were infected with *M. bovis* BCG at an MOI of 10 for 24 h or 48 h at 37 °C. Then the infected cells were lysed in 1 ml of sterile distilled water with 0.01% TritonX-100. Quantitative culturing was performed using 10-fold serial dilutions. Aliquots of each dilution were inoculated in triplicate on Middlebrook 7H10 agar plates supplemented with 10% OADC. After incubation for 3 weeks, colonies on plates were counted. The survival rate was calculated as compared to the control.

### Transient transfection of plasmids, siRNA, miRNA mimic or inhibitor

The cDNA sequence of murine TRAF6 was amplified by reverse transcription-PCR and cloned into pSG5 vector following the manufacturer’s protocol. Negative control (NC) and TRAF6 siRNAs were purchased from GenePharma (Shanghai, China). The target sequences of siRNA are as followings: siTRAF6-1, 5′-GCUACGAUGUGGAGUUUGAdTdT-3′; siTRAF6-2, 5′-GCGCUGUGCAAACUAUAUAdTdT-3′. RAW264.7 cells were transiently transfected with miR-146a mimic (30 nM) or inhibitor (50 nM) (RiboBio, Guangzhou, China), 1.6 μg plasmid or 100 nM siRNA, using Lipofectamine 2000 (Invitrogen) according to the manufacturer’s instructions.

### Real-time PCR

Total RNA was isolated using TRIzol reagent (Invitrogen) according to the manufacturer’s recommendation^50^,^51^. The expression of miR-146a was detected using a Bulge-Loop^TM^ miRNA qRT-PCR primer kit (RiBoBio, Guangzhou, China) and normalized to small nuclear RNA (U6). cDNAs were synthesized from 1 μg total RNA using RevertAid™ First Strand cDNA Synthesis Kit (Thermo Fisher Scientific, Waltham, MA). Quantitative real-time PCR analysis of TRAF6 and iNOS mRNA was performed on Bio-Rad CFX96 real-time detection system using SYBR Green Master Mix (Applied Biosystems, Foster City, CA). Primers used for real-time PCR amplification were as follows: β-actin, 5′-GATTACTGCTCTGGCTCCTAGC-3′ (forward), 5′-GACTCATCGTACTCCTGCTTGC-3′ (reverse); TRAF6, 5′- AAAGCGAGAGATTCTTTCCCTG-3′ (forward), 5′-ACTGGGGACAATTCACTAGAGC-3′ (reverse); iNOS, 5′-TCCTCACTGGGACAGCACAGAATG-3′ (forward), 5′-GTGTCATGCAAAATCTCTCCACTGCC-3′ (reverse).

### Western blot

Western blot was performed as described previously^52^,^53^,^54^. In brief, equal amounts of cell lysates were resolved by SDS-PAGE and then transferred to polyvinylidene fluoride or nitrocellulose membranes. Membranes were blocked and incubated overnight with primary antibodies at 4 °C. The membranes were incubated with appropriate HRP-conjugated secondary antibodies at room temperature for 1 h, and the blots were visualized with PlusECL (KeyGEN BioTECH) according to the manufacturer’s protocol. Alternatively, blots were detected with IRDye 800 CW conjugated anti-rabbit IgG or IRDye 680 CW conjugated anti-mouse IgG secondary antibodies (LI-COR Biosciences, Lincoln, NE), and visualized using Odyssey infrared imaging system (LI-COR Biosciences).

### Phagocytosis assay by flow cytometry

Phagocytosis of mycobacteria was examined by flow cytometry as described previously^26^. Briefly, *M. bovis* BCG was labeled with Texas Red dye for 2 h in dark with gentle shaking, and then rinsed with PBS according to the manufacturer’s instruction. RAW264.7 cells were challenged by the Texas Red-labeled BCG at an MOI of 10 for an hour. Then cells were washed thoroughly with cold PBS and centrifuged to remove extracellular bacteria. Cells were collected and analyzed using Beckman Coulter EPICS XL/MCL (Beckman Coulter Inc) as described before^49^.

### Measurement of NO production

NO production was determined by the amount of nitrite accumulated in the supernatant of cultured cells using a Griess reagent (Promega Corporation, Madison, WI) according to manufacturer’s protocol. Briefly, 50 μl of the supernatants were collected and mixed with 50 μl of Griess reagent consists of 0.1% naphtylethylenediamide dihydrochloride in H_2_O and 50 μl of 1% sulphanilamide in 5% H_2_PO_4_. The absorbance was measured at 550 nm, and nitrite concentration was calculated using a standard curve of sodium nitrite prepared in culture medium.

### Confocal microscopy

Confocal microscopy was performed as described previously^26^. Briefly, cells grown on cover slips were fixed with 4% paraformaldehyde followed by membrane permeabilization, blocking and antibodies incubation (anti-NF-κB p65, #8242, Cell Signaling Technology). Nuclei were stained with 4,6-diamidino-2-phenylindole (DAPI). Cover slips were mounted with ProLong Gold antifade reagent (Invitrogen) and viewed by confocal microscopy (Zeiss Axiovert, LSM710).

### Statistical analysis

Data were expressed as the mean ± s.e.m. of at least three independent experiments. Statistical analysis was performed using GraphPad Prism 5.0 (GraphPad Software, San Diego, CA). The results from real-time PCR, Griess assay and CFU assay were compared by Student’s *t* test or one-way analysis of variance with Bonferroni’s post-test. Differences were considered statistically significant with *p* < 0.05.

## Additional Information

**How to cite this article**: Li, M. *et al.* microRNA-146a promotes mycobacterial survival in macrophages through suppressing nitric oxide production. *Sci. Rep.* **6**, 23351; doi: 10.1038/srep23351 (2016).
