# Corrigendum: microRNA-146a promotes mycobacterial survival in macrophages through suppressing nitric oxide production

**DOI:** 10.1038/srep24555

**Published:** 2016-04-22

**Authors:** Miao Li, Jinli Wang, Yimin Fang, Sitang Gong, Meiyu Li, Minhao Wu, Xiaomin Lai, Gucheng Zeng, Yi Wang, Kun Yang, Xi Huang

Scientific Reports 6: Article number: 23351;10.1038/srep23351 published online: 03302016; updated: 04222016

This Article contains typographical errors in a grant number in the Acknowledgements section.

“This work was supported by grants National Natural Science Foundation of China (331470877, 81172811),”

should read:

“This work was supported by grants National Natural Science Foundation of China (31470877, 81172811),”

